# Detection of a New Resistance-Mediating Plasmid Chimera in a *bla*_OXA-48_-Positive *Klebsiella pneumoniae* Strain at a German University Hospital

**DOI:** 10.3390/microorganisms9040720

**Published:** 2021-03-31

**Authors:** Julian Schwanbeck, Wolfgang Bohne, Ufuk Hasdemir, Uwe Groß, Yvonne Pfeifer, Boyke Bunk, Thomas Riedel, Cathrin Spröer, Jörg Overmann, Hagen Frickmann, Andreas E. Zautner

**Affiliations:** 1 Institute for Medical Microbiology, University Medical Center Göttingen, 37075 Göttingen, Germany; julian.schwanbeck@med.uni-goettingen.de (J.S.); wbohne@gwdg.de (W.B.); ugross@gwdg.de (U.G.); 2 Department of Medical Microbiology, School of Medicine, Marmara University, 34854 Istanbul, Turkey; ufukhasdemir@yahoo.com; 3 Robert Koch Institute, FG13 Nosocomial Infections and Antibiotic Resistance, 38855 Wernigerode, Germany; PfeiferY@rki.de; 4 Leibniz Institute DSMZ-German Collection of Microorganisms and Cell Cultures, 38124 Braunschweig, Germany; boyke.bunk@dsmz.de (B.B.); thomas.riedel@dsmz.de (T.R.); ckc@dsmz.de (C.S.); joerg.overmann@dsmz.de (J.O.); 5 German Center for Infection Research (DZIF), Partner Site Hannover-Braunschweig, 38124 Braunschweig, Germany; 6 Department of Microbiology and Hospital Hygiene, Bundeswehr Hospital Hamburg, 20359 Hamburg, Germany; hagen.frickmann@med.uni-rostock.de; 7 Institute for Medical Microbiology, Virology and Hygiene, University Medicine Rostock, 18057 Rostock, Germany

**Keywords:** *Klebsiella pneumoniae*, carbapenem resistance, beta-lactamase, resistome, plasmid, phylogeny, epidemiology

## Abstract

Mobile genetic elements, such as plasmids, facilitate the spread of antibiotic resistance genes in Enterobacterales. In line with this, we investigated the plasmid-resistome of seven *bla*_OXA-48_ gene-carrying *Klebsiella pneumoniae* isolates, which were isolated between 2013 and 2014 at the University Medical Center in Göttingen, Germany. All isolates were subjected to complete genome sequencing including the reconstruction of entire plasmid sequences. In addition, phenotypic resistance testing was conducted. The seven isolates comprised both disease-associated isolates and colonizers isolated from five patients. They fell into two clusters of three sequence type (ST)101 and two ST11 isolates, respectively; and ST15 and ST23 singletons. The seven isolates harbored various plasmids of the incompatibility (Inc) groups IncF, IncL/M, IncN, IncR, and a novel plasmid chimera. All *bla*_OXA-48_ genes were encoded on the IncL/M plasmids. Of note, distinct phenotypical resistance patterns associated with different sets of resistance genes encoded by IncL/M and IncR plasmids were observed among isolates of the ST101 cluster in spite of high phylogenetic relatedness of the bacterial chromosomes, suggesting nosocomial transmission. This highlights the importance of plasmid uptake and plasmid recombination events for the fast generation of resistance variability after clonal transmission. In conclusion, this study contributes a piece in the puzzle of molecular epidemiology of resistance gene-carrying plasmids in *K. pneumoniae* in Germany.

## 1. Introduction

Plasmids play a major role as causative entities of acquired antimicrobial resistance in Gram-negative bacteria. In particular, horizontal spread of antimicrobial resistance is frequently driven by the conjugation-based transmission of plasmids [1]. Although the replication of resistance-mediating plasmids is associated with fitness costs for bacterial pathogens [2], compensatory mutations alleviate the associated evolutionary disadvantages [1,3]. Adaptive mutations in intergenic regions and selection of genes involved in anaerobic metabolism are thought to specifically stabilize the persistence of plasmid-carrying bacteria in the intestine of colonized individuals [4].

Due to the significant public health impact of plasmid-mediated resistance transfer, bioinformatic solutions for the identification of plasmid sequences from next generation sequence data obtained from bacterial pathogens with acquired resistances were introduced early on [5]. In particular, long read (PacBio) sequencing has proven to be a particularly suitable tool to completely resolve the DNA sequence of extrachromosomal mobile genetic elements like plasmids [6,7]. These methods can be used for the assessment of abundance, quantity, and diversity of plasmids in whole microbial communities.

In microbial species like *Escherichia coli* and *Klebsiella pneumoniae*, multidrug-resistance is mainly associated with acquisition and maintenance of plasmids encoding the resistance genes [4]. The geographic distribution patterns of plasmids suggests that special plasmid families are particularly successful in the global spreading of resistance genes. So-called epidemic resistance plasmids comprise plasmids of incompatibility (Inc) groups IncFII, IncA/C, IncL/M, IncN, and IncI1; these carry genes that encode for extended-spectrum β-lactamases (ESBLs), AmpC β-lactamases, and carbapenemases. All of these cephalosporin and carbapenem hydrolyzing enzymes have been globally identified in Enterobacterales of different sources and origins [8].

IncF plasmids have been reported to be associated with the global spread of *K*. *pneumoniae* producing CTX-M-15 ESBL [9,10], *Klebsiella pneumoniae* carbapenemases (KPCs) [11,12,13,14,15], and New Delhi metallo-β-lactamases (NDM) [16]. Their size and the number of replicons is heterogeneous. Replicon sequence typing has been applied for their further characterization [17]. IncL/M plasmids are associated with the carriage of *bla*_OXA-48_ carbapenemase genes [18,19]. Thereby, the *bla*_OXA-48_ gene has been reported to be specifically encoded on the IncL sequence [10,20]. Although IncL/M plasmids are predominantly prevalent in the Mediterranean region and Western Europe [21], their global spread has been confirmed by identifications even in tropical Brazil [22]. Moreover, IncN-plasmids have also been described to be widely distributed in *K. pneumoniae* [16,23,24] and to harbor resistance genes, such as *bla*_CTX-M-1_ [25], *bla*_KPC_ [26] and *bla*_IMP-6_ [27], respectively. Closely related to IncF plasmids, IncF/IncR-plasmids encoding *bla*_KPC_ have been reported [14]. Similar to IncF plasmids, IncR plasmids have also been associated with *bla*_KPC_ genes [28,29] and *bla*_NDM_ genes [30,31], among other resistance-mediating genes [32,33,34]. The *bla*_KPC_ gene-association has also been proven for plasmid chimerae with IncR-elements [35].

In 2013–2014, the nosocomial transmission of an oxacillinase-48 (OXA-48) carbapenemase producing *K. pneumoniae* strain of sequence type (ST)147 was detected at the University Medical Center Göttingen, Germany. During these investigations, seven further isolates of four different STs were identified [36]. Here, we characterize the plasmid-based acquired resistome of these seven *bla*_OXA-48_-carrying *K. pneumoniae* isolates. This explorative assessment will contribute a piece to the puzzle of local resistance epidemiology.

## 2. Materials and Methods

### 2.1. Bacterial Isolates and Clinical Information

Seven *bla*_OXA-48_-positive clinical *K. pneumoniae* isolates, which were isolated from five patients at the diagnostic laboratory of the University Medical Center Göttingen, Germany between 2013 and 2014, were included in the plasmid-resistome analysis. During a previous epidemiological assessment, multilocus sequence typing (MLST) assigned three isolates from two patients to sequence type ST101, two isolates from one patient to ST11, and two isolates from two patients to ST23 and ST15 [36]. Clinical information provided for the otherwise fully anonymized isolates comprised patient sex, patient age, site of isolation, and underlying medical condition (Table 1).

### 2.2. Species Identification and Resistance

Bacterial species identification was performed using the MALDI (Matrix-Assisted Laser Desorption/Ionization) Biotyper system (Bruker Daltonics, Bremen, Germany). Results with MALDI Biotyper identification score values ≥ 2.000 were assessed as correct.

Antimicrobial susceptibilities to 17 antibiotics (piperacillin, piperacillin/tazobactam, cefepime, aztreonam, cefotaxime, ceftazidime, imipenem, meropenem, gentamicin, amikacin, tobramycin, trimethoprim-sulfamethoxazole, colistin, fosfomycin, ciprofloxacin, moxifloxacin, and tigecycline) were assessed using VITEK 2 card AST N248 (bioMérieux, Hilden, Germany). Interpretation was performed according to the recommendations of the European Committee on Antimicrobial Susceptibility Testing (EUCAST) breakpoints version v11.0 (http://www.eucast.org/clinical_breakpoints, last accessed on 10 February 2021). Transfer of resistance was tested in broth mating experiments; the sodium azide-resistant strain *E. coli* J53 Azi^r^ served as the recipient. Transconjugants were selected on LB agar plates containing sodium azide (200 mg/L), ampicillin (30 mg/L), and a disk with imipenem (10 µg). Antimicrobial susceptibilities and presence of β-lactamase genes were tested, and plasmid content and sizes were determined by S1-nuclease restriction and pulsed-field gel electrophoresis (PFGE) as described before [37].

### 2.3. Whole Genome Sequencing and Bioinformatics

The seven isolates were identified as carbapenemase producers that harbored carbapenemase gene *bla*_OXA-48_, and genome sequence information was generated as described previously [36]. In brief, DNA extractions were subjected to Single-molecule real-time (SMRT) sequencing on a PacBio *RSII* (Pacific Biosciences, Menlo Park, CA, USA). Using the same DNA preparation, short-read sequencing was performed on a HiSeq 2500 device (Illumina Inc., San Diego, CA, USA). SMRT cell data were assembled independently using the RS_HGAP_Assembly.3 protocol. Briefly, each replicon was circularized independently and the artificial redundancies at the ends of the contigs were removed. The validity of the assembly was checked using the RS_Bridgemapper.1 protocol. Each genome was corrected for indel errors by a mapping of Illumina short-reads onto the SMRT long-read assembled genomes. A consensus concordance of QV60 could be confirmed for all genomes. The assembled genomes were deposited at GenBank. The accession numbers are listed in Table 3 and Table A1. Assembled plasmid contigs were subjected to BLASTN search at the National Center for Biotechnology Information (https://blast.ncbi.nlm.nih.gov/Blast.cgi, accessed on 17 June 2019). The annotation of the assembled genomes was performed applying ‘rapid annotations using subsystems technology’ (RAST) (http://rast.nmpdr.org, accessed on 17 June 2019). Annotated genomes were scanned in the SEED viewer (https://seed-viewer.theseed.org/, accessed on 5 June 2016). Spreadsheet charts with protein coding genes (CDS) were obtained and analyzed for antibiotic resistance genes (ARGs) and mobile genetic elements (MGEs). In addition, the completely assembled genomes in fasta format were provided as inputs to the web-based ResFinder 2.1 tool (http://cge.cbs.dtu.dk/services/ResFinder/, accessed on 22 December 2016) and ARGs identities were accepted at an increased identity threshold of >96% and a min length of 60% [36].

### 2.4. Plasmid Visualization

Alignment results from BLASTN were visualized using Kablammo (http://kablammo.wasmuthlab.org/, accessed on 15 February 2021), with minimal bit score set to 2000. Single plasmid maps were generated using Geneious Prime v2021.0.3 (Biomatters Ltd., Auckland, New Zealand).

## 3. Results

### 3.1. Clinical Information

The seven *K. pneumoniae* isolates were from five male patients between 31 and 53 years of age. The isolates comprised etiologically relevant isolates from urine in case of urinary tract infection (*n* = 2), from tracheal secretion in case of pneumonia (*n* = 1) and from wounds in case of wound infections (*n* = 2). Furthermore, two ST11 isolates were mere colonizers as detected by hygiene-related routine swabbing (Table 1). Of note, the two isolates from patient 3 were isolated in a temporal distance of about one month. Core genome MLST that was performed in a previous study [36] revealed close genetic relationship of the three ST101 isolates and the two ST11 isolates, respectively.

### 3.2. Antibiotic Resistance Assessment and Transferability

**Figure A1:**
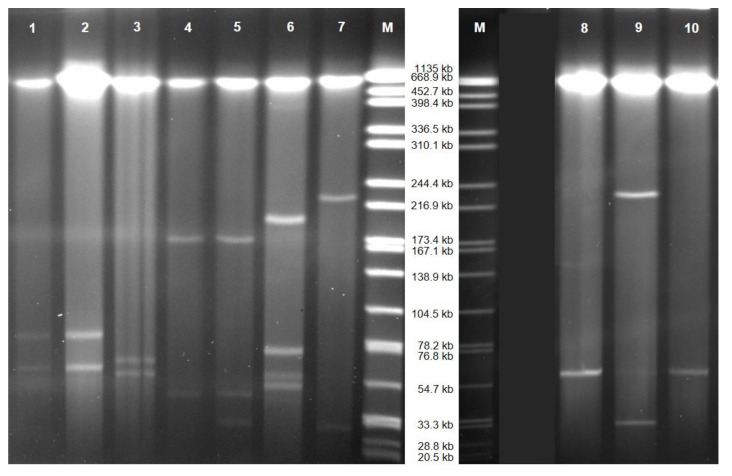
Plasmids of seven OXA-48 producing *Klebsiella pneumoniae* isolates and two *Escherichia coli* transconjugants visualized by S1-nuclease restriction and pulsed-field gel electrophoresis. Lane 1, *K. pneumoniae*-ST101 Kp_Goe_33208; lane 2, *K. pneumoniae*-ST101 Kp_Goe_71070; lane 3, *K. pneumoniae*-ST101 Kp_Goe_121641; lane 4, *K. pneumoniae*-ST11 Kp_Goe_821588; lane 5, *K. pneumoniae*-ST11 Kp_Goe_822917; lane 6, *K. pneumoniae*-ST23 Kp_Goe_154414; lane 8, Kp_Goe_121641-*E. coli* J53 transconjugant *bla*_OXA-48_-positive; lanes 7 and 9, *K. pneumoniae*-ST15 Kp_Goe_39795; lane 10, Kp_Goe_39795-*E. coli* J53 transconjugant *bla*_OXA-48_-positive; lane M, *Salmonella enterica,* serotype Braenderup H9812 (*Xba*I-restricted).

All seven isolates were resistant to piperacillin, piperacillin/tazobactam, aztreonam, cefotaxime, ceftazidime, gentamicin, tobramycin, ciprofloxacin, moxifloxacin, and trimethoprim-sulfamethoxazole. Resistance and reduced susceptibility to imipenem (range between 0.5 and >8 mg/L) and meropenem (range between 1 and >8 mg/L) was detected for all seven isolates, and the two ST11 isolates were additionally resistant to colistin (Table 2). For two isolates (Kp_Goe_121641—ST101 and Kp_Goe_39795—ST15) the broth mating experiment were successful. The obtained transconjugants were positive for *bla*_OXA-48_, were resistant to piperacillin and MICs of 1–2 mg/L were detected for imipenem and meropenem. S1-nuclease pulsed field gel electrophoresis (PFGE) showed the presence of a plasmid of ca. 60 kb size in the transconjugants (Appendix B Figure A1).

### 3.3. Analysis of the Plasmids and Comparison with Phenotypical Resistance

**Figure 1:**
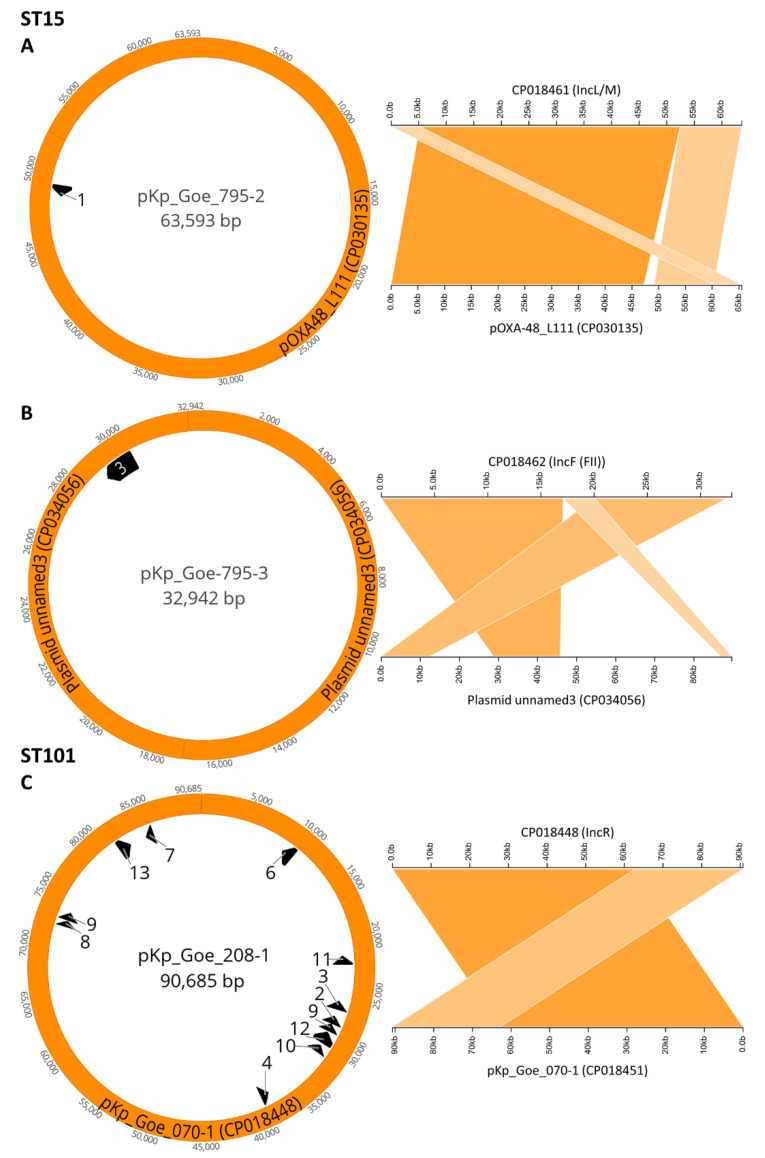
Example plasmids found in OXA-48 producing *Klebsiella pneumoniae* of sequence types ST15 (**A**,**B**) and ST101 (**C**). Plasmids are mapped to possible origins found by National Center for Biotechnology Information (NCBI) BLAST as described in Appendix A Table A1. For ST15 (**A,B**): (**A**) Plasmid pKp_Goe_795-2 (CP018461, from Kp_Goe_39795) is mapped to the reference plasmid pOXA-48_L111 (CP030135, query coverage 100%, identity 100%). (**B**) Plasmid pKp_Goe-795-3 (CP018462, from Kp_Goe_39795) is mapped to the reference plasmid “unnamed3” (CP034056, query coverage 100%, identity 99.98%). (**C**) For ST101, pKp_Goe208-1 (CP018448, from Kp_Goe_33208,) is mapped to the reference plasmid pKp_Goe_070-1 (CP018451, query coverage 100%, identity 99.99%). Resistance genes as predicted by ResFinder: 1: *bla*_OXA_-_48_, 2: *bla*_OXA_-_9_, 3: *bla*_TEM_-_1A_, 4: *bla*_TEM_-_1C_, 5: *tetA*, 6: *tetD*, 7: *aac*(3)-IIa, 8: *aac*(6′)-Ib, 9: *aadA*1, 10: *aadA*2b, 11:*mphA*, 12: *cmlA*1, 13: *floR*.

**Figure 2:**
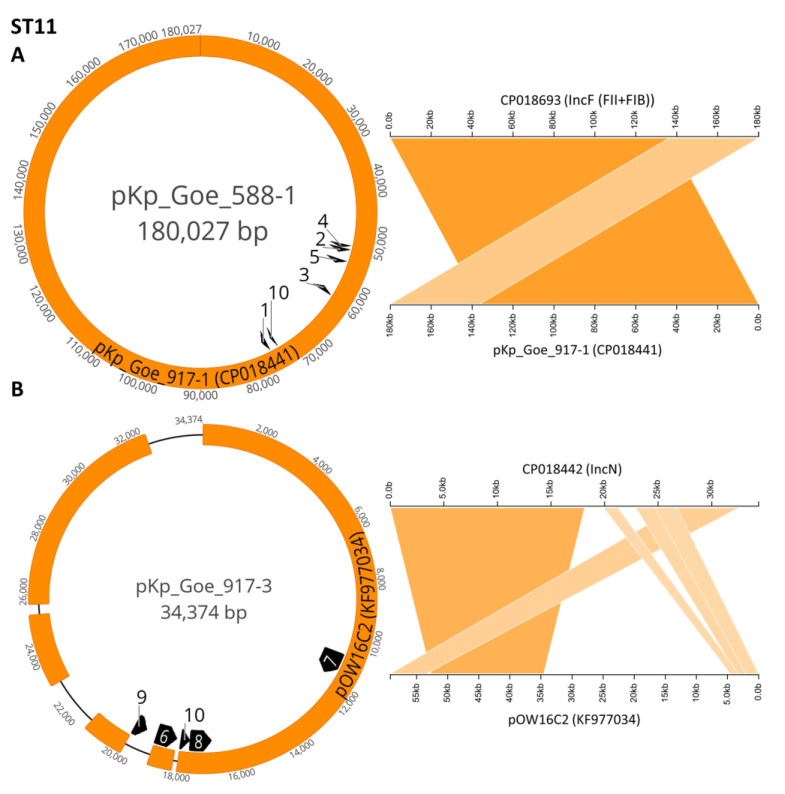
Example Plasmids found in OXA-48 producing *Klebsiella pneumoniae* isolates of sequence types ST11. Plasmids are mapped to possible origins found by NCBI BLAST as described in Appendix A Table A1. (**A**) Plasmid pKp_Goe_588-1 (CP018693, from Kp_Goe_821588) is mapped to pKp_Goe_917-1 (CP018441, query coverage 100%, identity 100%). (**B**) pKp_Goe_917-3 (CP018442, from Kp_Goe_822917) is mapped to pOW16C2 (KF977034, query coverage 92%, identity 99.99%). Resistance genes as predicted by ResFinder: 1: *aph*(3′)-Ia, 2: *bla*_OXA_-_1_, 3: *bla*_CTX_-_M-15_, 4: *aac*(6′)-Ib-cr, 5: *aac*(3)-IIa, 6: *aadA*2, 7:*mphA*, 8: *sul*1, 9: *dfrA*12, 10: *dfrA*14, 11: *qacE*.

**Figure 3:**
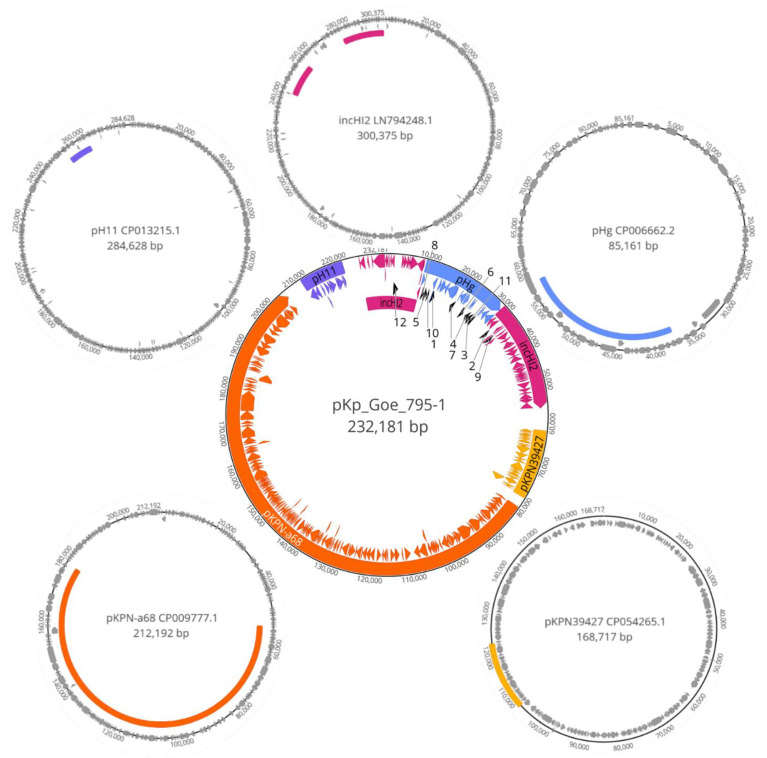
Plasmid chimaera pKp_Goe_795-1 and possible origins. Plasmid pKp_Goe_795-1 (CP018460) was isolated from strain Kp_Goe_39795 of sequence type ST15. Mapped regions had an identity of >98% with correspondingly colored areas from putative origins and a minimal length of 10 kbp. Regions found include plasmids pHg (CP006662), pKPN39427 (CP054265), pKPN-a68 (CP009777), pH11 (CP013215), as well as two disjointed regions from plasmid incHI2 (LN794248). Resistance genes as predicted by ResFinder: 1: *aac*(3)-IIa, 2: *ant*(3″)-Ia, 3: *aph*(3″)-Ib, 4: *aph*(6)-Id, 5: *bla*_OXA_-_1_, 6: *bla*_TEM_-_1B_, 7: *bla*_CTX_-_M-15_, 8: *aac*(6′)-Ib-cr, 9: *catA*1, 10: *catB*3, 11: *sul*2, 12: *tetA*.

As visualized in Figure 1, Figure 2 and Figure 3, and in Table 3, numerous resistance genes occurred in various types of plasmids of the lineages IncL/M, IncR, IncF, and IncN. Each isolate had at least two different types of plasmids, one of which was IncL/M with *bla*_OXA-48_ (example: Figure 1A).

**Figure 4:**
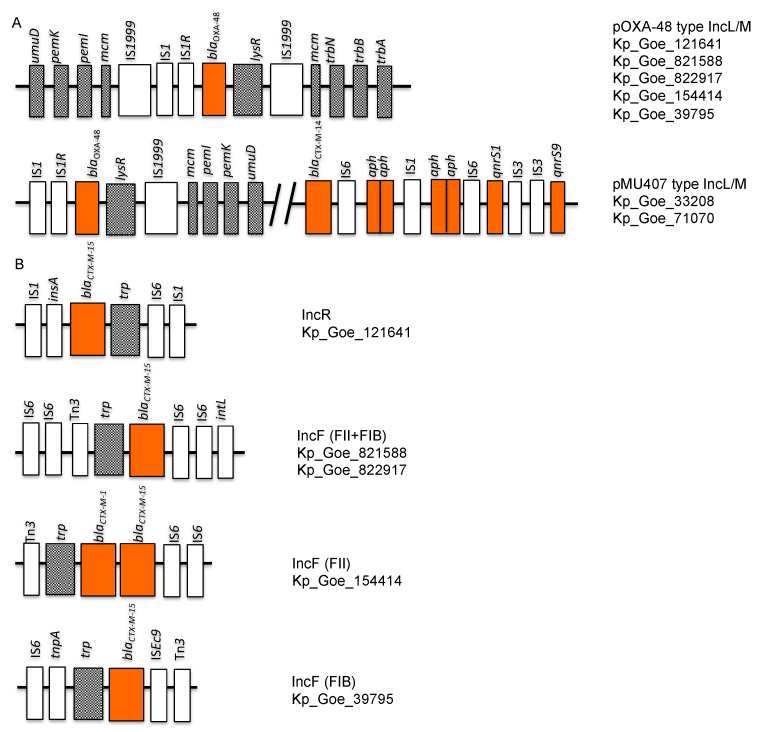
Summary of the genetic synteny of *bla*_OXA-48_, *bla*_CTX-M-14_, and *bla*_CTX-M-15_ genes on the plasmids of *Klebsiella pneumoniae* isolates. (**A**) the locations of *bla*_OXA-48_, *bla*_CTX-M-14_, and other antimicrobial resistance genes on the pMU407 and pOXA-48 type IncL/M plasmids (**B**) the locations of *bla*_CTX-M-15_ genes on the IncR and IncF type plasmids.

The resistance gene *bla*_OXA-48_, whose abundance was the selection criterion of the seven isolates for this study, was exclusively associated with IncL/M plasmids of variable size; plasmid pKp_Goe_795-2 of ST15 isolate Kp_Goe_39795 is shown exemplarily in Figure 1A. These plasmids, however, further hosted additional antibiotic resistance genes (ARGs) besides *bla*_OXA-48_ gene in two out of three isolates of ST101 (Table 3). In five isolates (Kp_Goe_121641, Kp_Goe_821588, Kp_Goe_822917, Kp_Goe_154414, Kp_Goe_39795), the IncL/M plasmids showed 100% identity with the reference plasmid pOXA-48 (GenBank accession number: JN626286). Complete sequences of these plasmids were similar to each other above the 99.9% level, but were also similar to some other plasmids in enterobacterial species collected from the same region and other countries (Appendix A Table A1). In contrast, the IncL/M plasmids of the two ST101 isolates from patient 1 (Kp_Goe_33208 and Kp_Goe_71070) showed 99.59% identity with the *K. pneumoniae* plasmid pMU407 (GenBank accession number: U27345). The identity between the complete sequences of these two plasmids was 100%. In contrast to the third ST101 isolate from patient 2, further resistance genes (e.g., ESBL gene *bla*_CTX-M-14b_ and plasmid mediated quinolone resistance determinants *qnrS1/9*) were located on the IncL/M plasmid with *bla*_OXA-48_ (Table 3, Figure 4). The comparative BLAST analysis of complete sequences also revealed 100% identity with a 90% query coverage to a plasmid (GenBank accession number: KP025948) from a *Proteus mirabilis* strain (Appendix A Table A1) [38]. 

**Figure 5:**
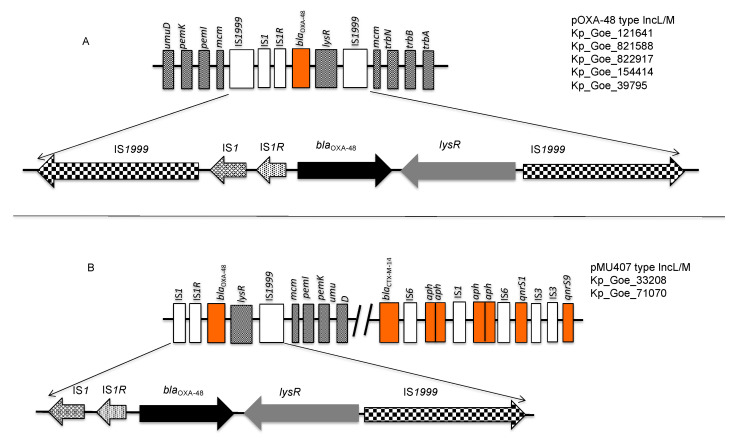
Comparison of transposons encoding OXA-48. (**A**) In five of the tested isolates, the *bla*_OXA-48_ gene was part of Tn*1999*; (**B**) in the two ST101 isolates Kp_Goe_33208 and Kp_Goe_71070 the *bla*_OXA-48_ gene was part of a Tn*1999.2* transposon, which is characterized by the insertion of IS*1R* into the upstream IS*1999* region.

Analysis of the genetic synteny of the *bla*_OXA-48_ gene revealed its location within an element of transposon Tn*1999.2* in the two IncL/M plasmids of ST101 isolates (Kp_Goe_33208 and Kp_Goe_71070). The *bla*_OXA-48_ gene was flanked by *lysR* and IS*1999*, respectively, downstream and by IS*1R* and IS*1* upstream. On the other hand, McmM, TrbN, TrbB, and TrbA encoding genes were found downstream of Tn*1999* in pOXA-48 type IncL/M plasmids of the other five isolates (Figure 5).

As indicated in Figure 3 and Table 3, the ST15 isolate Kp_Goe-39795 carried a large 232kb plasmid chimera that has not been described before. This IncF plasmid (Figure 3) with FIB replicon did not show similarity with any known plasmid in BLAST analysis. It encoded the ARGs *bla*_CTX-M-15_, *bla*_OXA-1_, *bla*_TEM-1_, *aac*(6′)-Ib-cr, *aac*(3)-IIa, *aph*(6)-Id, *aph*(3″)-Ib, *ant*(3″)-Ia, *catB3*, *catA1*, *sul2*, *tetA*, as well as various heavy metal resistance determinants. The mobile genetic elements flanked by these genes are shown in Table 3. Individual parts of this plasmid chimera could be matched to putative origin plasmids (Figure 3). GenBank accession numbers of all detected plasmids in the seven *K. pneumoniae* isolates are shown in Appendix A Table A1.

In four out of seven isolates, IncF plasmids were present (Table 3); these carried various genes that mediate resistance to β-lactams, aminoglycosides, phenicols, tetracyclines, sulfonamides and trimethoprim. The presence of these genes corresponded with the observed phenotypes. Examples for reconstructed IncF plasmids are given in Figure 1B and Figure 2A. IncFII, IncFIB, and IncFIA replicons were detected on these plasmids (Appendix A Table A1). The complete sequences of the IncF plasmids of the two ST11 isolates Kp_Goe_821588 and Kp_Goe_822917 were identical to each other. These plasmids carried both FII and FIB replicons, resistance genes *bla*_CTX-M-15_, *bla*_OXA-1_*, aac*(6′)-Ib-cr, *aac*(3)-IIa, *aph*(3′)-Ia, *catB3*, *dfr*A14, and genes mediating resistance to heavy metals, such as arsenic, cobalt, zinc, cadmium and copper (Table 3). Kp_Goe_154414 (ST 23) had four IncF type plasmids. Three out of them harbored several ArGs encoding resistance to β-lactams, aminoglycosides, quinolones, phenicols, sulfonamides, tetracycline, and heavy metals (Table 3).

In three ST101 isolates (Kp_Goe_33208 (Figure 1C), Kp_Goe_71070, and Kp_Goe_121641), IncR plasmids carried a composition of ARGs similar to those found on the IncF plasmids, one example is given in Figure 1B. The backbones of these plasmids were 100% identical with the plasmid pK245 (Gene accession number: DQ449578). The complete sequences of the IncR plasmids of isolates from patient 1 (Kp_Goe_33208 and Kp_Goe_71070) were identical to each other. Their identity with the smaller IncR plasmid of Kp_Goe_121641 was 99.99% with a coverage of 74%. The resistance genes *cat*, *ant*(3″)-*Ia, aac(6′)-Ib, aac(6′)-Ib-cr, aac(3)-IIa, bla*_TEM-1A_, *bla*_OXA-9_, *dfrA14, merE*, *merT*, *merC* were detected in all IncR plasmids (Table 3). In addition to this, *tetA*, *tetD*, *mph(A)*, *emrE, sul3, cmlA,* and *floR* were located on the IncR plasmids of the isolates Kp_Goe_33208 and Kp_Goe_71070 compared to Kp_Goe_121641 (Table 3). The additional ArGs on the IncR plasmid of isolate Kp_Goe_121641 included β-lactamase genes *bla*_CTX-M-15_ and *bla*_OXA-1_. The *bla*_CTX-M-15_ gene was flanked downstream by *insA* and IS*1* and upstream by the tryptophan synthase coding gene and IS*1* (Figure 4B).

An IncN plasmid was detected in one of the two *K. pneumoniae*-ST11 isolates from patient 3 (Kp_Goe_822917), associated with a number of genes that mediate resistance to macrolides, trimethoprim and ethidium bromide (Figure 2B, Table 3).

## 4. Discussion

The study was performed to contribute to the existing epidemiological knowledge on the distribution of antibiotic resistance-mediating plasmids in carbapenemase producing *K. pneumoniae* isolates in Germany. The characterized seven isolates represent epidemic clonal lineages of *K. pneumoniae* (ST11, ST15, ST101, and ST23) that have been described worldwide and are associated with multidrug resistance and/or enhanced virulence [39]. The genetic relationship of these bacterial isolates has been first characterized in detail in a previous study [36]. To allow an unambiguous attribution of the described plasmids to the previously described bacterial isolates, the specific identifier codes Kp_Goe_xxxxx are identical in the previous manuscript [36] and in the present one.

As expected, the so-called epidemic resistance plasmids IncF, IncL/M, and IncN [8] that carry various resistance-mediating genes were identified in these seven isolates. Furthermore, we identified IncR plasmids and a new IncF (FIB) plasmid chimera.

As reported by others, the *bla*_OXA-48_ genes waere associated with IncL/M plasmids [10,18,19,20]. As typical for *bla*_OXA-48_, which is only associated with high-level carbapenem resistance in case of combination with other resistance mediating elements [18], the majority of the seven *K. pneumoniae* isolates were phenotypically tested susceptible towards carbapenems (Table 2).

The tracking of carbapenem resistance-associated mechanisms remains an issue of relevance, as infections, due to carbapenem-resistant *K. pneumoniae,* are globally reported, and are rising [40,41,42]. Thereby, plasmid-encoded carbapenemase production is the major mechanism of carbapenem resistance in *K. pneumoniae*. While mobile genetic elements such as plasmids, transposons, and insertion sequences readily enable the transmission of carbapenemase-encoding genes, clonal expansion contributes to the globally increasing rates of dissemination of carbapenemase-producing *K. pneumoniae* [40,41,42]. Next to transmission events within human microbiomes, spread of resistance determinants may also occur in external environments under the selection pressure of pollution with antimicrobial active substances [43].

Our seven study isolates, which included both disease-associated and merely colonizing isolates, were associated with the carriage of resistance-mediating plasmids. As shown for the phylogenetically closely related isolates of the sequence type ST101 (confirmed by cgMLST [36]) indicating likely nosocomial transmission, their accessory genome varied remarkably. Cause of such variance is a different set of resistance mediating plasmids, encoding various resistance determinants (Table 3) that may create phenotypical variations. Discrepancies in phenotypical resistance does therefore not necessarily exclude nosocomial spread. However, isolates from the same patients showed identical phenotypic resistance in our study, although variations in plasmid content were detected for two ST11 isolates (Table 3).

While carbapenem resistance, or at least reduced carbapenem susceptibility compared to the wild type, was associated with *bla*_OXA-48_ in all cases, this resistance gene is not the only one that has been frequently detected in *K*. *pneumoniae*. In the early 2000s, three types of carbapenemases, *Klebsiella pneumoniae* carbapenemase (KPC), oxacillinase-48 (OXA-48) type carbapenemase, and the New Delhi metallo-β-lactamase (NDM), emerged globally and spread rapidly. Nosocomial outbreaks due to carbapenemase producing *K. pneumoniae* strains became an alarming issue in hospital setting in many countries in Europe and worldwide [40,41,42]. To cite some examples, IncF plasmids with the IncFII_K_ replicon mediated the global spread of KPC-encoding genes throughout the United States, Colombia, Argentina, Israel, Greece, Norway, Sweden, Italy, Poland, Canada, Brazil, Korea, and Taiwan [40,41,42]. Starting at the Indian subcontinent, New Delhi metallo-β-lactamase producing *K. pneumoniae* have rapidly spread to various countries such as Romania, Poland, Hungary, Denmark, Italy, Spain, Greece, Turkey, China, Australia, Japan, Colombia, South Africa, Algeria, Morocco, Saudi Arabia, and Oman [40,41,42] in association with the plasmids IncA/C, IncFII, IncN, IncH, and IncL/M. In contrast, OXA-48 producing *K. pneumoniae*, such as those examined in the presented study, were first identified in Turkey in 2001 and showed a rapid global dissemination [40,41,42,44]. Just as shown for the ST11 isolates in our study, *bla*_OXA-48_, encoding the OXA-48 carbapenemase, was first identified as a part of Tn*1999* on an IncL/M plasmid, pOXA-48a (GenBank accession number JN626286). As observed for the isolates presented here, the global dissemination of *bla*_OXA-48_ in *K. pneumoniae* has been mainly linked to the epidemic IncL/M plasmid [10,18,19,20]. The variant of Tn*1999*, Tn*1999.2*, which was observed in two of the analyzed ST101 isolates, has been detected in *K. pneumoniae* carrying *bla*_OXA-48_ together with *bla*_CTX-M-14-b_ and other antimicrobial resistance genes, while other authors reported the association of *bla*_OXA-48_ together with *bla*_CTX-M-15_ [45]. While the IncL/M plasmid was the genetic element primarily responsible for reduced carbapenem susceptibility or carbapenem resistance in our isolates, the other detected IncF, IncN, and IncR plasmids carried various resistance genes and contributed to their multidrug-resistance phenotype (Table 3).

The observation of the new plasmid chimera pKp_Goe_795-1 (CP018460, Figure 3) is of particular interest, as it documents “real life evolution” by the assembly of resistance determinants in one, and the same plasmid vector through extensive transposition. Associated with this process, it is worth focusing on the epidemiological background of transposases and other ARG-associated mobile genetic elements (MGEs) found in the genetic information of the plasmid chimera (Table 3). The reported IS*6* insertion sequence family has previously been reported from a multidrug resistance-mediating plasmid in *Proteus mirabilis* in China [46]. IS*6* also allowed migration of a replicative elements carrying ARGs by replicative transposition in *Klebsiella pneumoniae* in France and in Japan [47,48]. Tn*3*-like transposons have recently been reported from Enterobacterales including *K. pneumoniae* from China and Switzerland [49,50,51]. The transition protein-encoding *tnp1* has been described from *E. coli* in China [52]. The insertion sequence IS*1380* has been first identified in *Acinetobacter pasteurianus* [53,54], the insertion sequence IS*110* was recently reported from *Klebsiella pneumoniae* from New Jersey [55], IS*1* in *K. pneumoniae* from China and Russia [56,57], IS*481* in *K. pneumoniae* from Japan [48], IS*903* in *K. pneumoniae* from France and China [58,59], and in *Salmonellae* from the USA [60]. IS*66* is abundant in the worldwide-distributed *K. pneumoniae* sequence types ST258/ST512 [61]. IS*3*, using a two-step transposition mechanism to specifically insert into short palindromic repeated sequences, has been reported from *K. pneumoniae* in Europe and the USA [62,63,64]. IntIPac was described from a IncFIB plasmid found in a clinical *Klebsiella variicola* isolate in Hong Kong [65]. A new member of the insertion sequence family ISNCY has been reported from *K. pneumoniae* in California, USA, some years ago [63]. The insertion sequence IS*L3* has been linked to the transposition of sequence information for colistin resistance in Europe [66] and for carbapenem resistance in China [67] in *K. pneumoniae*. Summarized, the observed MGEs are internationally common in Enterobacterale*s* in general and *K. pneumoniae* in particular, while just the arrangement in the newly described plasmid has not yet been described so far.

The limited number of assessed isolates is the major limitation of this study presented here. Nevertheless, the presented data provide a piece in the puzzle of molecular epidemiology of resistance-mediating plasmids in Germany. It contributes to previous efforts to improve our understanding how *bla*_OXA-48_ and other antimicrobial resistance genes spread among *K. pneumoniae* isolates [36,68,69].

## 5. Conclusions

The study demonstrated the abundance of various IncF, IncM/L, IncN, and IncR plasmids in seven OXA-48 producing *K. pneumoniae* isolates from hospitalized patients in Germany. Long read sequencing enabled the complete plasmid reconstruction and identification of a novel IncF (FIB) plasmid chimera. It further highlighted the association of *bla*_OXA-48_ with IncL/M plasmids in *K. pneumoniae*. This information, once implemented in bioinformatics tools for molecular identification of antibiotic resistance, will help to improve the molecular epidemiology of resistance plasmids and resistance genes. Thus, it contributes to the prediction of phenotypic susceptibilities and improve our understanding of the evolution of resistance gene-encoding plasmids. Future challenges for the goal of achieving broad surveillance of resistance-mediating MGEs comprise ready and affordable availability of sequencing technology and lacking automation of bioinformatic assessments, which is still associated with an unrealistically high work-load for application under routine-diagnostic conditions. Further declines of sequencing costs and increased implementation of artificial intelligence (AI) solutions in order to reduce the required hands-on time of experts may guide the way to achieve molecular near-real-time surveillance in the future.

## Figures and Tables

**Table 1:** Clinical information available for the seven assessed oxacillinase-48 (OXA-48) producing *bla*_OXA-48_-positive *Klebsiella pneumoniae* isolates from five hospitalized patients in Germany from the blinded analyses. Isolates from the same patient are shaded in identical gray shades.

Sample-ID	Sequence Type (ST)	Patient No. and Sex	Patient Age at Isolation	Date of Isolation	Sampling Location	Underlying Medical Condition as Attributed to the Isolate
Kp_Goe_33208	ST101	1, Male	31	2013-04-05	Wound at the perianal location	Wound infection
Kp_Goe_71070	ST101	1, Male	31	2013-04-05	Urine	Urinary tract infection
Kp_Goe_121641	ST101	2, Male	32	2013-03-12	Urine	Urinary tract infection
Kp_Goe_821588	ST11	3, Male	50	2014-02-11	Anal region	Hygiene assessment (no disease association)
Kp_Goe_822917	ST11	3, Male	50	2013-03-12	Hairline on the forehead	Hygiene assessment (no disease association)
Kp_Goe_154414	ST23	4, Male	36	2014-07-21	Wound at the hand	Accident-related surgical intervention at the hand
Kp_Goe_39795	ST15	5, Male	53	2014-09-23	Tracheal secretion	Pneumonia

**Table 2:** Antibiotic susceptibilities of seven OXA-48 producing *Klebsiella pneumoniae* isolates.

Sample ID	Patient No.	ST	PIP	TZP	CEF	ATM	CTX	CAZ	IPM	MEM	GEN	AMK	TOB	CIP	MOX	TIG	CST	FOS	SXT
Kp_Goe_33208	1	101	>64	>64	>32	>32	>32	>32	>8	>8	>8	>32	>8	>2	>4	1	≤0.5	≤16	>160
Kp_Goe_71070	1	101	>64	>64	>32	>32	>32	>32	>8	>8	>8	>32	>8	>2	>4	1	≤0.5	≤16	>160
Kp_Goe_121641	2	101	>64	>64	>32	>32	>32	>32	2	1	>8	16	>8	>2	>4	≤0.5	≤0.5	≤16	40
Kp_Goe_821588	3	11	>64	>64	>32	>32	>32	>32	4	4	>8	8	>8	>2	>4	2	>8	32	40
Kp_Goe_822917	3	11	>64	>64	>32	>32	>32	>32	2	2	>8	4	>8	>2	>4	2	>8	≤16	40
Kp_Goe_154414	4	23	>64	>64	>32	>32	>32	>32	4	>8	>8	4	>8	>2	>4	2	≤0.5	128	80
Kp_Goe_39795	5	15	>64	>64	2	>32	>32	16	0.5	1	>8	≤2	>8	>2	>4	>4	≤0.5	64	40

Gray shading: resistant. All measured minimum inhibitory concentrations are given in mg/L. Detected resistances (EUCAST v11.0; http://www.eucast.org/clinical_breakpoints, last accessed on 2 December 2021) are shaded in grey. ST, sequence type; PIP, piperacillin; TZP, piperacillin/tazobactam; CEF, cefepime, ATM, aztreonam, CTX, cefotaxime, CAZ, ceftazidime; IPM, imipenem; MEM, meropenem; GEN, gentamicin; AMK, amikacin; TOB, tobramycin; CIP, ciprofloxacin; MOX, moxifloxacin; TIG, tigecycline CST, colistin; FOS, fosfomycin; SXT, trimethoprim-sulfamethoxazole.

**Table 3:** Detected plasmids, associated antibiotic resistance genes, and detected antibiotic resistances in seven OXA-48 producing *Klebsiella pneumoniae* isolates.

Sample ID	DSM No. ^a^	Accession Number	City	MLST ^b^	Plasmid Type	Plasmid Size (Base Pairs)	Antibiotic Resistance Genes (ARG)	ARG-Associated Mobile Genetic Elements (MGE)	Detected Resistance Phenotype
Kp_Goe_33208	DSM 103696	CP018449	Seesen	ST101	IncL/M	67,101	*bla*_OXA-48_	Tn*1999.2*	piperacillin, piperacillin/tazobactam, aztreonam, cefotaxime, ceftazidime, gentamicin, tobramycin, amikacin, ciprofloxacin, and moxifloxacin, imipenem, meropenem, amikacin, trimethoprim-sulfamethoxazole
							*bla*_CTX-M-14b_, *qnrS1*, *qnrS9*, *aph(6)-Id*, *aph(3′)-VI*, *aph(3″)*	IS*6*, IS*1*, IS*3* families	
		CP018448			IncR	90,685	*bla*_TEM-1A_, *bla*_TEM-1C_, *bla*_OXA-9_, *aac(6′)-Ib*, *aac(6′)-Ib-cr*, *aac(3)-IIa*, *ant(3″)-Ia*, *tetA*, *tetD*, *mph(A)*, *cat*, *cmlA*, *floR*, *dfr*A14, *sul3*, *emrE*, *merE*, *merT*, *merC*	IS*6*, IS*1*, Tn*3*, Integron IntI pac, IS*256*, TnpA, IS*3*	
Kp_Goe_71070	DSM 103699	CP018452	Seesen	ST101	IncL/M	67,100	*bla*_OXA-48_	Tn*1999.2*	piperacillin, piperacillin/tazobactam, aztreonam, cefotaxime, ceftazidime, gentamicin, tobramycin, amikacin, ciprofloxacin, and moxifloxacin, imipenem, meropenem, amikacin, trimethoprim-sulfamethoxazole
							*bla*_CTX-M-14b,_ *qnrS1*, *qnrS9*, aph*(6)-Id*, *aph(3′)-VI*, *aph(3″)*	IS*6*, IS*1*, IS*3* families	
		CP018451			IncR	90,684	*bla*_TEM-1A_, *bla*_TEM-1C_, *bla*_OXA-9_, *aac(6′)-Ib*, *aac(6′)-Ib-cr*, *aac(3)-IIa*, *ant(3″)-Ia*, *tetA*, *tetD*, *mph(A)*, *cat*, *cmlA*, *floR*, *dfr*A14, *sul3*, *emrE*, *merE*, *merT*, *merC*	IS*6*, IS*1*, Tn*21*, Tn*3*, Integron IntI pac, IS*256*, TnpA, IS*3*	
Kp_Goe_121641	DSM 103707	CP018736	Göttingen	ST101	IncL/M	63,589	*bla*_OXA-48_	Tn*1999*, IS*1*, IS*4* families	piperacillin, piperacillin/tazobactam, aztreonam, cefotaxime, ceftazidime, gentamicin, tobramycin, amikacin, ciprofloxacin, moxifloxacin, trimethoprim-sulfamethoxazole
		CP018737			IncR	72,952	*bla*_CTX-M-15_, *bla*_OXA-1_, *bla*_OXA-9_, *bla*_TEM-1A_, *ant(3″)-Ia*, *aac(6′)-Ib*, *aac(6′)-Ib-cr*, *aac(3)-IIa*, *dfr*A14, *cat*, *merE*, *merT*, *merC*	IS*6*, IS*1* (IS1 family ISEcp1 element), Tn*21*, Tn*3*, IS*256*, IS*3*	
Kp_Goe_821588	DSM 103700	CP018694	Göttingen	ST11	IncL/M	50,609	*bla*_OXA-48_	Tn*1999*, IS*1*, IS*4* families	piperacillin, piperacillin/tazobactam, aztreonam, cefotaxime, ceftazidime, gentamicin, tobramycin, ciprofloxacin, moxifloxacin, trimethoprim-sulfamethoxazole, colistin
		CP018693			IncF (FII + FIB)	180,027	*bla*_CTX-M-15_, *bla*_OXA-1_, *aac(6′)-Ib-cr*, *aac(3)-IIa*, *aph(3′)-Ia*, *aac(3′)-III* *catB3*, *dfr*A14 Heavy metal (arsenic, copper, cobalt, cobalt, zinc, cadmium) resistance determinants*^d^*	IS*903*, Tn*3*, IS*6*, Integron integrase IntIpac, IS*5* (IS*1182*-DUF772)	
Kp_Goe_822917	DSM 103702	CP018443	Göttingen	ST11	IncL/M	50,611	*bla*_OXA-48_	Tn*1999*, IS*1*, IS*4* families	piperacillin, piperacillin/tazobactam, aztreonam, cefotaxime, ceftazidime, gentamicin, tobramycin, ciprofloxacin, moxifloxacin, trimethoprim-sulfamethoxazole, colistin
		CP018441			IncF (FII + FIB)	180,027	*bla*_CTX-M-15_, *bla*_OXA-1_, *aac(6′)-Ib-cr*, *aac(3)-IIa*, *aph(3′)-Ia*, *aac(3′)-III* *catB3*, *dfr*A14 Heavy metal (arsenic, copper, cobalt, cobalt, zinc, cadmium) resistance determinants*^d^*	IS*903*, Tn*3*, IS*6*, Integron integrase IntIpac, IS*5* (IS*1182*-DUF772)	
		CP018442			IncN	34,374	*ant(3″)-Ia*, *dfrA(12)*, *sul1*, *mph(A)*, *tetR*, *emrE*	Tn*3*, IS*6*	
Kp_Goe_154414	DSM 103711	CP018342	Göttingen	ST23	IncL/M	63,588	*bla*_OXA-48_	Tn*1999*, IS1, IS4 families	piperacillin, piperacillin/tazobactam, aztreonam, cefotaxime, ceftazidime, gentamicin, tobramycin, ciprofloxacin, and moxifloxacin, meropenem, Fosfomycin, trimethoprim-sulfamethoxazole
		CP018338			Inc(FIB)	202,175	Heavy metal (copper, cobalt, cobalt, zinc, cadmium, tellurium) resistance determinants, RND efflux protein	IS*5*, Tn*3*, IS*110*, IS*3*, IS*1*, Tn*21*, IS*630*, IS*21*, IS*66*	
		CP018343			IncF(FII)	57,226	*bla*_CTX-M-55_, *bla*_OXA-1_, *aac(3)-IIa*, *aac(6′)-Ib-cr*, *catB3*	Tn*3*, IS*6*, IS*66*, IS*1*, Tn*1721* (Tn*3*), IS*3*	
		CP018341			IncFII_K_	81,641	class A β-lactamase (LAP family), *qnrS1*, *catA2*, *sul2*, *tetA*	IS*3*, Tn*3*, IS*6*, IS*110* le (IS*5075*), IS*91* (TnpA)	
		CP018340			IncF(FIA)	81,939			
Kp_Goe_39795	DSM 103697	CP018461	Seesen	ST15	IncL/M	63,593	*bla*_OXA-48_	Tn*1999*, IS*1*, IS*4* families	piperacillin, piperacillin/tazobactam, aztreonam, cefotaxime, ceftazidime, gentamicin, tobramycin, ciprofloxacin, and moxifloxacin, tigecycline, fosfomycin, trimethoprim-sulfamethoxazole
		CP018460			IncF (FIB)	232,181	*bla*_CTX-M-15_, *bla*_OXA-1_, *bla*_TEM-1B_ *aac(6′)-Ib-cr*, *aac(3)-IIa*, *aph(6)-Id*, *aph(3″)-Ib*, *ant(3″)-Ia*, *catB3*, *catA1*, *sul2*, *tetA*, *tetR*, Heavy metal (arsenic, copper, cobalt, cobalt, zinc, cadmium) resistance determinants*^d^*	IS*6*, Tn*3*, Tnp*1* (IS*1380* le), IS*110* le, Integron integrase IntIPac, IS*1*, IS*NCY* le, IS*L3*, IS*66*, IS*3*, IS*481* le, IS*903*	
		CP018462			IncF(FII)	32,942	*tetA*, *tetR*	Tn*3* (Tn*1721*), Tn*3* le, IS*6*	

^a^ deposited at the Leibniz Institute DSMZ-German Collection of Microorganisms and Cell Cultures GmbH (Deutsche Sammlung von Mikroorganismen und Zellkulturen GmbH); ^b^ Multilocus sequence typing (MLST).

## Data Availability

All relevant data are provided in the paper. The bacterial genomes have been deposited at NCBI GenBank and can be accessed via the accession numbers given in the article.

## Appendix A

**Table A1:** GenBank Accession numbers and BLAST results of the plasmids of *Klebsiella pneumoniae* isolates on the basis of PlasmidFinder-2.0 of Center for Genomic Epidemiology (CGE) and National Center for Biotechnology Information (NCBI).

			Plasmid Finder		NCBI		
	Plasmid	Accession No.	Identity (%)	Identity with (Accession No.)	Query Coverage (%)	Identity (%)	Identity with Plasmid (Accession No.)
Kp_Goe_33208	IncL/M	CP018449	99.59	pMU407.1 (U27345)	90	100	pOXA-48-pm (KP025948)
					87	100	pEC743 (CP015071)
					84	98.85	pKpvST383L (CP034202)
					83	99.94	pJEG011 (KC354801)
	IncR	CP018448	100	pK245 (DQ449578)	79	99.94	unnamed plasmid (CP032176)
					74	100	pKp_Goe_641-1 (CP018737)
					100	99.99	pKp_Goe_070-1 (CP018451)
Kp_Goe_71070	IncL/M	CP018452	99.59	pMU407.1 (U27345)	90	100	pOXA-48-pm (KP025948)
					87	100	pEC743 (CP015071)
					84	98.85	pKpvST383L (CP034202)
					83	99.94	pJEG011 (KC354801)
	IncR	CP018451	100	pK245 (DQ449578)	79	99.92	unnamed plasmid (CP032176)
					74	99.99	pKp_Goe_641-1 (CP018737)
					100	100	pKp_Goe_208-1 (CP018448)
Kp_Goe_121641	IncL/M	CP018736	100	pOXA-48 (JN626286)	100	100	pOXA-48_A12536 (LR025100)
					100	100	pOXA-48_E7215 (LR025098)
					100	100	pOXA-48_980 (LR025091)
					100	100	pOXA-48_920 (LR025095)
					>99	>99.91	pKp_Goe_579_1, pKp_Goe_832-1, pKp_Goe_414-5, pKp_Goe_024, pKp_Goe_795-2, pKp_Goe_021-1, pKp_Goe_026-1
	IncR	CP018737	100	pK245 (DQ449578)	91	100	pKp_Goe_208-1 (CP018448)
					91	99.99	pKp_Goe_070-1 (CP018451)
					58	100	pKPN101-IT (JX283456)
Kp_Goe_821588	IncL/M	CP018694	100	pOXA-48 (JN626286)	100	99.98	pOXA-48_L111 (CP030135)
					100	99.98	pKPN-E1.Nr7 (KM406491)
					100	99.95	pEC745_OXA-48 (CP015075)
					100	100	Kp_Goe_121641, Kp_Goe822917, Kp_Goe_154414
					100	99.99	pKp_Goe_795-2 (CP018461.1)
					100	100	Kp_Goe_152021, Kp_Goe_827026, Kp_Goe_149473, Kp_Goe_828304, Kp_Goe_822917, Kp_Goe_827024, Kp_Goe_149832, Kp_Goe_822579
	IncF (FII + FIB)	CP018693	100	pKPN3 (CP000648)	100	100	pKp_Goe_917-1 (CP018441)
					94	100	plasmid IncFIB IncFII (CP027037)
					91	100	plasmid p1 (CP006657)
Kp_Goe_822917	IncL/M	CP018443	100	pOXA-48 (JN626286)	100	100	Kp_Goe_821588, Kp_Goe_154414, Kp_Goe_39795
					100	100	Kp_Goe_152021, Kp_Goe_149473, Kp_Goe_827026, Kp_Goe_828304, Kp_Goe_827024, Kp_Goe_149832, Kp_Goe_822579
							plasmid IncLM (CP027039)
	IncF (FIB + FII)	CP018441	100 (FIB)	pKPN-IT (JN233704)	100	100	CP018693, Kp_Goe_821588
			100 (FII)	pKPN3 (CP000648)	94	100	plasmid IncFIB IncFII (CP027037)
					91	100	plasmid p1 (CP006657)
					93	99.98	pKp_Goe_629-1 (CP018365)
	IncN	CP018442			92	99.99	plasmid pOW16C2 (KF977034)
					90	97.72	pNL194 (GU585907)
Kp_Goe_39795	IncL/M	CP018461	100	pOXA-48 (JN626286)	100	99.99	pOXA-48_L111 (CP030135)
					100	99.97	pKPN-E1.Nr7 (KM406491)
					100	100	pKp_Goe_121641, pKp_Goe_821588
					100	100	pKp_Goe_828304, pKp_Goe_149473, pKp_Goe_152021, pKp_Goe_827026,
	IncF (FIB_K_)	CP018460	100	pKPN3 (JN233704)	-	-	New (this study)
	IncF(FII)	CP018462	100	pC15-1a (AY458016)	82	99.98	pKp_Goe_414-1 (CP018143)
					100	99.98	Plasmid unnamed3 (CP034056)
					100	99.97	pEK516 (EU935738)
					100	99.98	pC15-1a (AY458016)
Kp_Goe_154414	IncL/M	CP018342	100	pKPN3 (JN233704)	100	100	Plasmid unnamed2 (CP032174)
					100	100	pOXA-48 (LR025105)
					100	100	pOXA-48 (LR025091)
					100	99.99	pKp_Goe_641-2 (CP018736)
					100	99.99	pKp_Goe_827024, pKp_Goe_149832, pKp_Goe_822579, pKp_Goe_827026, pKp_Goe_152021, pKp_Goe_149473, pKp_Goe_828304
	IncF(FIB)	CP018338	99.54	pNDM-MAR (JN420336)	95	99.88	plasmid F81 (CP026166)
					92	99.76	plasmid L22-1 (CP031258)
					100	99.99	pVir_095132 (CP028390)
					100	99.99	Goe-827024, 149832, 822579, 827026, 152021, 149473, 828304,121641
	IncF(FII)	CP018343	100	pC15-1a (AY458016)	95	99.83	*K. pneumoniae* KP_NORM_BLD_2015_112126 plasmid unnamed3 (CP034056)
					84	99.99	*K. pneumoniae* M16-13 plasmid pM16-13 (KY751925)
					95	99.99	*K. pneumoniae* strain NY9 plasmid pNY9_3 (CP015388)
	IncFII_K_	CP018341	98.65	pKPN3 (CP000648)	100	99.97	*K. pneumoniae* p4-L388, ST11 KPC-1 producer, 2018 China (CP029223)
					100	99.97	*K. pneumoniae* pQnr-S1_020079, 2018 China (CP029382)
					98	99.97	*K. pneumoniae* plasmid p3s1, 2018, China (CP034126)
	IncF(FIA)	CP018340	97.16	R27 plasmid (AF250878)	83	99.37	pEC25-1 (CP035124)
					81	99.37	pQnrB (CP025966)
					81	99.37	pKPC2_095132 (CP028389)
